# RECRUITMENT OF PERSONS WITH COGNITIVE IMPAIRMENT FROM LONG-TERM CARE SETTINGS

**DOI:** 10.1093/geroni/igad104.2915

**Published:** 2023-12-21

**Authors:** Rebecca Davis, Alla Sikorskii

**Affiliations:** Grand Valley State University, Grand Rapids, Michigan, United States; Michigan State University, East Lansing, Michigan, United States

## Abstract

Research that includes persons with cognitive impairment is important to develop evidence-based care to improve outcomes. Long -term care settings have a large proportion of residents with cognitive impairment; but recruitment is known to be difficult. The purpose of this study was to compare the effectiveness of recruitment methods for a randomized controlled clinical trial of persons with cognitive impairment in 15 long-term care communities. This study utilized a retrospective, descriptive design. Sites were recruited and site characteristics recorded. Residents were recruited to participate in the study based on combinations of 12 recruitment strategies. Recruitment data was analyzed at the level of site, research staff member, and participant level. Over the study period, 279 older adults were screened and 172 enrolled. The participants were cognitively impaired. Research staff spent 39-89 hours recruiting at each site and utilized an average of four different recruitment methods per site. The most common and efficacious method was introductions of participants by site contacts. Cost of recruiting averaged $1490 per site and was correlated to the number of resident rooms (r=.60, p=0.17). Those sites with a recruiter enrolled more participants (M=14.67, S.D. 4.55) than those without a recruiter (M=9.3, S.D. 2.55), t(13) = -2.92, p=.012. Recruitment of older adults with cognitive impairment from long term care communities requires multiple methods of recruitment; skilled recruitment staff, and trust with the LTC community staff and potential participants.

